# 2-*n*-Butyl-6-chloro-1-(2,4-dimethyl­phenyl­sulfon­yl)-1*H*-benzimidazole–2-*n*-butyl-5-chloro-1-(2,4-dimethyl­phenyl­sulfon­yl)-1*H*-benzimidazole (0.759/0.241)

**DOI:** 10.1107/S1600536811047957

**Published:** 2011-11-19

**Authors:** K. B. Abdireymov, N. S. Mukhamedov, R. Ya. Okmanov, M. J. Ayimbetov, B. Tashkhodjaev

**Affiliations:** a S. Yunusov Institute of the Chemistry of Plant Substances, Academy of Sciences of Uzbekistan, Mirzo Ulugbek Str., 77, Tashkent 100170, Uzbekistan; b Kara-Kalpak State University, Acad. Abdirov Str., 1, Nukus 742000, Uzbekistan

## Abstract

The title compound, 0.759C_19_H_21_ClN_2_O_2_S·0.241C_19_H_21_ClN_2_O_2_S, was synthesized by aryl­sulfonyl­ation of 2-*n*-butyl-5-chloro-1*H*-benzimidazole in the presence of triethyl­amine. The crystal structure is composed of two mol­ecules, 2-*n*-butyl-6-chloro-1-(2,4-dimethylphenyl­sulfon­yl)-1*H*-benzimidazole and 1-(2,4-dimethylphenyl­sulfon­yl)-2-*n*-butyl-5-chloro-1*H*-benz­imidazole, in the refined ratio of 0.759 (4):0.241 (4) disordered at the same position in the unit cell. The mol­ecule has three essentially planar fragments *viz.* benzimidazole, dimethyl­benzene and *n*-butyl (r.m.s. deviations of 0.009, 0.024 and 0.003 Å, respectively). The angle between the benzimidazole and dimethyl­benzene fragments is 86.0 (1)°. In the crystal, pairs of inter­molecular C—H⋯π inter­actions form centrosymmetrical dimers, which are linked by weak inter­molecular C—H⋯O hydrogen bonds.

## Related literature

For the biological and pharmaceutical properties of benz­imid­azole derivatives, see: Koči *et al.* (2002 ▶); Matsuno *et al.* (2000 ▶); Garuti *et al.* (1999 ▶). For the synthesis, biological activity and related structures of 2-*n*-butyl­benzimidazole derivatives, see: Kubo *et al.* (1993*a* ▶,*b* ▶); For the aryl­sulfonyl­ation of benzimidazole derivatives, see: Abdireimov *et al.* (2010 ▶). 
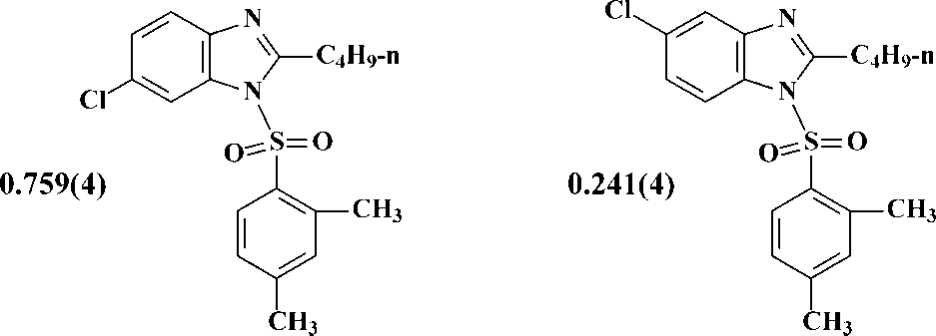


## Experimental

### 

#### Crystal data

0.759C_19_H_21_ClN_2_O_2_S·0.241C_19_H_21_ClN_2_O_2_S
*M* *_r_* = 376.89
Triclinic, 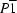

*a* = 8.7340 (17) Å
*b* = 10.251 (2) Å
*c* = 11.390 (2) Å
α = 71.29 (3)°
β = 78.38 (3)°
γ = 76.75 (3)°
*V* = 931.1 (3) Å^3^
*Z* = 2
Cu *K*α radiation
μ = 2.98 mm^−1^
*T* = 290 K
0.68 × 0.45 × 0.20 mm

#### Data collection

Stoe Stadi-4 four-circle diffractometer
Absorption correction: ψ scan (*X-RED*; Stoe & Cie, 1997 ▶) *T*_min_ = 0.250, *T*_max_ = 0.551
2722 measured reflections
2714 independent reflections
2460 reflections with *I* > 2σ(*I*)
θ_max_ = 60.0°
3 standard reflections every 60 min  intensity decay: 10.4%

#### Refinement

*R*[*F* ^2^ > 2σ(*F* ^2^)] = 0.069
*wR*(*F* ^2^) = 0.191
*S* = 1.12
2714 reflections
240 parameters
H-atom parameters constrained
Δρ_max_ = 0.35 e Å^−3^
Δρ_min_ = −0.45 e Å^−3^

### 

Data collection: *STADI4* (Stoe & Cie, 1997 ▶); cell refinement: *STADI4*; data reduction: *X-RED* (Stoe & Cie, 1997 ▶); program(s) used to solve structure: *SHELXS97* (Sheldrick, 2008 ▶); program(s) used to refine structure: *SHELXL97* (Sheldrick, 2008 ▶); molecular graphics: *XP* in *SHELXTL* (Sheldrick, 2008 ▶); software used to prepare material for publication: *publCIF* (Westrip, 2010 ▶).

## Supplementary Material

Crystal structure: contains datablock(s) I, global. DOI: 10.1107/S1600536811047957/im2330sup1.cif

Structure factors: contains datablock(s) I. DOI: 10.1107/S1600536811047957/im2330Isup2.hkl

Additional supplementary materials:  crystallographic information; 3D view; checkCIF report

## Figures and Tables

**Table 1:** Hydrogen-bond geometry (Å, °) *Cg*3 is the centroid of the C12–C17 ring.

*D*—H⋯*A*	*D*—H	H⋯*A*	*D*⋯*A*	*D*—H⋯*A*
C19—H19*B*⋯O2^i^	0.96	2.62	3.554 (7)	165
C4—H4*A*⋯*Cg*3^ii^	0.93	2.76	3.665 (8)	163

Symmetry codes: (i) 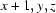; (ii) 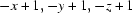.

**Fig. 1.**
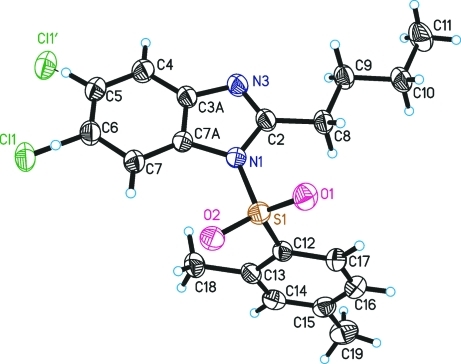
Molecular structure of the title compound, displacement ellipsoids are drawn at the 30% probability level.

## supplementary crystallographic information

### Comment

Benzimidazole (Koči *et al.*, 2002; Matsuno *et al.*, 2000; Garuti *et al.*, 1999) and 2-*n*-butylbenzimidazole (Kubo *et al.*, 1993*a*; 1993*b*) derivatives are important heterocyclic compounds which have attracted great attention due to their biological and pharmaceutical activities.

Reaction of 2-1*n*-butyl-5-chloro-1*H*-benzimidazole with 2,4-dimethylbenzenesulfonyl chloride in the presence of triethylamine results in a mixture of 1-(2,4-dimethylbenzenesulfonyl)-2-*n*-butyl-6-chloro-1*H*-benzimidazole and 1-(2,4-dimethylbenzenesulfonyl)-2-*n*-butyl-5-chloro-1*H*-benzimidazole, in the refined ratio of 0.759 (4):0.241 (4) (Abdireimov *et al.*, 2010). The structure of the received product is investigated by ^1^H NMR and X-ray diffraction.

As a whole the molecule consists of three flat fragments: benzimidazole (N1/C2/N3/C3A–C7A), dimethylbenzene (C12–C19) and *n*-butyl (C8–C11) (r.m.s. deviations are 0.009, 0.024 and 0.003 Å, respectively). The angle between flat benzimidazole and dimethylbenzene fragment is 86.0 (1)°, and between benzimidazole and *n*-butyl is 4.4 (2)° (Fig. 1).

The crystal structure is stabilized by intermolecular C—H···π interactions observed between the atoms of two benzene rings of neighboring molecules with distance C4—H···*Cg*3^i^ = 3.665 (2) Å [symmetry code: (i) 1 - *x*, 1 - *y*, 1 - *z*; *Cg*3 is centroid of the C12–C17 benzene ring]. Observable C—H···π interactions form centrosymmetric dimers, another weak intermolecular H-bond such as C19—H···O2 sew these dimers (Table 1).

### Experimental

In the three-necked round-bottomed flask, supplied with a mechanical mixer, dropping funnel and backflow condenser, were placed 2.04 g (10 mmol) 2,4-dimethylbenzenesulfonyl chloride in 15 ml of acetone and was added a solution of 2.08 g (10 mmol) 2-1*n*-butyl-5-chloro-1*H*-benzimidazole and 1.01 g (10 mmol) triethylamine in 30 ml acetone by stirring and cooling. The reaction mixture was stirred at room temperature for 4 h. Afterwards acetone is evaporated. The residual product was washed with 100 ml of the water, obtained crystals were filtered and recrystallized from ethanol. 2.18 g (56%) mixed crystals of 1-(2,4-dimethylbenzenesulfonyl)-2-*n*-butyl-6-chloro-1*H*-benzimidazole (A) and 1-(2,4-dimethylbenzenesulfonyl)-2-*n*-butyl-5-chloro-1*H*-benzimidazole (B), melting in the temperature range of 108–117°C were obtained.

Colorless crystals suitable for XRD have been received from ethanol at room temperature.

### Refinement

The 10.4% decay correction was applied by using the programm *X-RED*. The H atoms bonded to C atoms were placed geometrically (with C—H distances of 0.97 Å for CH_2_; 0.96 Å for CH_3_; and 0.93 Å for C_ar_) and included in the refinement in a riding motion approximation with *U*_iso_=1.2*U*_eq_(C) [*U*_iso_=1.5*U*_eq_(C) for methyl H atoms].

### Crystal data

**Table:**

0.7590.241C_19_H_21_ClN_2_O_2_S·0.2410.241C_19_H_21_ClN_2_O_2_S	*Z* = 2
*M**_r_* = 376.89	*F*(000) = 396
Triclinic, *P*1	*D*_x_ = 1.344 Mg m^−^^3^
Hall symbol: -P 1	Melting point < 381(9) K
*a* = 8.7340 (17) Å	Cu *K*α radiation, λ = 1.54184 Å
*b* = 10.251 (2) Å	Cell parameters from 13 reflections
*c* = 11.390 (2) Å	θ = 10–20°
α = 71.29 (3)°	µ = 2.98 mm^−^^1^
β = 78.38 (3)°	*T* = 290 K
γ = 76.75 (3)°	Prizmatic, colorless
*V* = 931.1 (3) Å^3^	0.68 × 0.45 × 0.20 mm

### Data collection

**Table:**

Stoe Stadi-4 four-circle diffractometer	2460 reflections with *I* > 2σ(*I*)
Radiation source: fine-focus sealed tube	*R*_int_ = 0.000
graphite	θ_max_ = 60.0°, θ_min_ = 4.1°
Scan width (ω) = 1.56 – 1.80, scan ratio 2θ:ω = 1.00 I(Net) and sigma(I) calculated according to Blessing (1987)	*h* = −9→9
Absorption correction: ψ scan (*X-RED*; Stoe & Cie, 1997)	*k* = −10→11
*T*_min_ = 0.250, *T*_max_ = 0.551	*l* = 0→12
2722 measured reflections	3 standard reflections every 60 min
2714 independent reflections	intensity decay: 10.4%

### Refinement

**Table:**

Refinement on *F*^2^	Secondary atom site location: difference Fourier map
Least-squares matrix: full	Hydrogen site location: inferred from neighbouring sites
*R*[*F*^2^ > 2σ(*F*^2^)] = 0.069	H-atom parameters constrained
*wR*(*F*^2^) = 0.191	*w* = 1/[σ^2^(*F*_o_^2^) + (0.0919*P*)^2^ + 1.1929*P*] where *P* = (*F*_o_^2^ + 2*F*_c_^2^)/3
*S* = 1.12	(Δ/σ)_max_ < 0.001
2714 reflections	Δρ_max_ = 0.35 e Å^−^^3^
240 parameters	Δρ_min_ = −0.45 e Å^−^^3^
0 restraints	Extinction correction: *SHELXL*, Fc^*^=kFc[1+0.001xFc^2^λ^3^/sin(2θ)]^-1/4^
Primary atom site location: structure-invariant direct methods	Extinction coefficient: 0.026 (3)

### Special details

**Table:**

Experimental. Empirical absorption correction using ψ Scan. Reflections used Mu * R = 0.00 H K L, θ, χ, I_min_/I_max_: -1 -2 4 45.0 82.7 0.455^1^H NMR (400 MHz, CDCl~3~): 1-(2,4-dimethylbenzenesulfonyl)-2-*n*-butyl-6- chloro-1*H*-benzimidazole (A). 7.97 (1*H*, d, J═2.1 Hz, H-7), 7.49 (1*H*, d, J═8.3 Hz, H-17), 7.49 (1*H*, d, J═8.5 Hz, H-4), 7.37 (2*H*, d, J═7.9 Hz, H-14, 16), 7.29 (1*H*, dd, J═2.o, J═8.5 H-5), 3.08 (2*H*, m, CH_2_-8), 2.34 (6*H*, s, CH_3_-18, 19), 1.73 (2*H*, m, CH_2_-9), 1.37 (2*H*, m, CH_2_-10), 0.89 (3*H*, t, J═7.3 Hz, CH_3_-11).1-(2,4-dimethylbenzenesulfonyl)-2-*n*-butyl-5-chloro- 1*H*-benzimidazole (B). 7.95 (1*H*, d, J═8.7 Hz, H-7), 7.78 (1*H*, d, J═8.3 Hz, H-17), 7.52 (1*H*, d, J═2.0 Hz, H-4), 7.35 (2*H*, d, J═7.9 Hz, H-14, 16), 7.31 (1*H*, dd, J═2.o, J═8.5 H-6), 3.08 (2*H*, m, CH_2_-8), 2.34 (6*H*, s, CH_3_-18, 19), 1.73 (2*H*, m, CH_2_-9), 1.37 (2*H*, m, CH_2_-10), 0.90 (3*H*, t, J═7.5 Hz, CH_3_-11).
Geometry. All e.s.d.'s (except the e.s.d. in the dihedral angle between two l.s. planes) are estimated using the full covariance matrix. The cell e.s.d.'s are taken into account individually in the estimation of e.s.d.'s in distances, angles and torsion angles; correlations between e.s.d.'s in cell parameters are only used when they are defined by crystal symmetry. An approximate (isotropic) treatment of cell e.s.d.'s is used for estimating e.s.d.'s involving l.s. planes.
Refinement. Refinement of *F*^2^ against ALL reflections. The weighted *R*-factor *wR* and goodness of fit *S* are based on *F*^2^, conventional *R*-factors *R* are based on *F*, with *F* set to zero for negative *F*^2^. The threshold expression of *F*^2^ > σ(*F*^2^) is used only for calculating *R*-factors(gt) *etc*. and is not relevant to the choice of reflections for refinement. *R*-factors based on *F*^2^ are statistically about twice as large as those based on *F*, and *R*- factors based on ALL data will be even larger.

### Fractional atomic coordinates and isotropic or equivalent isotropic displacement parameters (Å^2^)

**Table:**

	*x*	*y*	*z*	*U*_iso_*/*U*_eq_	Occ. (<1)
S1	0.25613 (12)	0.65554 (11)	0.19565 (9)	0.0537 (4)	
O1	0.2520 (4)	0.5625 (3)	0.1267 (3)	0.0660 (9)	
O2	0.1230 (4)	0.7622 (3)	0.2077 (3)	0.0647 (9)	
N1	0.2783 (4)	0.5558 (3)	0.3424 (3)	0.0511 (8)	
N3	0.3767 (4)	0.3796 (4)	0.4993 (3)	0.0606 (10)	
C2	0.3863 (5)	0.4310 (4)	0.3785 (4)	0.0552 (10)	
C3A	0.2567 (5)	0.4705 (4)	0.5479 (4)	0.0552 (10)	
C4	0.1997 (6)	0.4606 (5)	0.6731 (4)	0.0688 (13)	
H4A	0.2428	0.3874	0.7370	0.083*	
C5	0.0780 (6)	0.5624 (5)	0.6986 (4)	0.0658 (12)	
H5A	0.0377	0.5584	0.7815	0.079*	0.759 (4)
C6	0.0142 (5)	0.6701 (5)	0.6052 (4)	0.0604 (11)	
H6A	−0.0684	0.7372	0.6269	0.072*	0.241 (4)
C7	0.0675 (5)	0.6835 (5)	0.4791 (4)	0.0573 (11)	
H7A	0.0226	0.7564	0.4158	0.069*	
C7A	0.1909 (5)	0.5817 (4)	0.4545 (4)	0.0498 (10)	
C8	0.4990 (5)	0.3662 (5)	0.2876 (4)	0.0606 (11)	
H8A	0.5704	0.4300	0.2385	0.073*	
H8B	0.4397	0.3519	0.2306	0.073*	
C9	0.5963 (6)	0.2267 (5)	0.3510 (4)	0.0640 (12)	
H9A	0.6586	0.2409	0.4061	0.077*	
H9B	0.5255	0.1631	0.4016	0.077*	
C10	0.7063 (7)	0.1625 (5)	0.2552 (5)	0.0754 (14)	
H10A	0.7774	0.2261	0.2052	0.090*	
H10B	0.6439	0.1496	0.1997	0.090*	
C11	0.8036 (7)	0.0226 (6)	0.3163 (6)	0.0918 (18)	
H11A	0.8725	−0.0141	0.2528	0.138*	
H11B	0.7337	−0.0417	0.3638	0.138*	
H11C	0.8662	0.0349	0.3710	0.138*	
C12	0.4289 (5)	0.7259 (4)	0.1379 (4)	0.0498 (10)	
C13	0.4696 (5)	0.8270 (4)	0.1800 (4)	0.0553 (11)	
C14	0.6081 (6)	0.8746 (4)	0.1239 (4)	0.0603 (11)	
H14A	0.6356	0.9413	0.1512	0.072*	
C15	0.7100 (5)	0.8296 (5)	0.0291 (4)	0.0615 (11)	
C16	0.6664 (6)	0.7320 (5)	−0.0118 (4)	0.0657 (12)	
H16A	0.7317	0.6998	−0.0757	0.079*	
C17	0.5288 (6)	0.6823 (5)	0.0405 (4)	0.0609 (11)	
H17A	0.5009	0.6178	0.0105	0.073*	
C18	0.3722 (6)	0.8773 (5)	0.2876 (5)	0.0702 (13)	
H18A	0.4075	0.9581	0.2906	0.105*	
H18B	0.3850	0.8043	0.3646	0.105*	
H18C	0.2623	0.9014	0.2761	0.105*	
C19	0.8634 (6)	0.8809 (7)	−0.0233 (6)	0.0881 (16)	
H19A	0.9019	0.8631	−0.1029	0.132*	
H19B	0.9399	0.8329	0.0331	0.132*	
H19C	0.8471	0.9796	−0.0340	0.132*	
Cl1	−0.1412 (2)	0.79798 (18)	0.64266 (16)	0.0782 (7)	0.759 (4)
Cl1'	0.0475 (7)	0.5668 (7)	0.8540 (5)	0.081 (2)	0.241 (4)

### Atomic displacement parameters (Å^2^)

**Table:**

	*U*^11^	*U*^22^	*U*^33^	*U*^12^	*U*^13^	*U*^23^
S1	0.0575 (7)	0.0540 (7)	0.0473 (6)	−0.0039 (5)	−0.0118 (5)	−0.0129 (5)
O1	0.077 (2)	0.073 (2)	0.0594 (18)	−0.0196 (16)	−0.0128 (15)	−0.0283 (16)
O2	0.0621 (18)	0.0621 (18)	0.0554 (18)	0.0096 (14)	−0.0143 (14)	−0.0076 (14)
N1	0.056 (2)	0.0460 (18)	0.0433 (18)	−0.0003 (15)	−0.0038 (15)	−0.0104 (15)
N3	0.068 (2)	0.053 (2)	0.054 (2)	0.0043 (17)	−0.0096 (17)	−0.0153 (17)
C2	0.056 (2)	0.049 (2)	0.059 (3)	0.0009 (19)	−0.0074 (19)	−0.020 (2)
C3A	0.058 (2)	0.050 (2)	0.054 (2)	0.0005 (19)	−0.0065 (19)	−0.0173 (19)
C4	0.082 (3)	0.066 (3)	0.049 (3)	0.000 (2)	−0.008 (2)	−0.013 (2)
C5	0.073 (3)	0.070 (3)	0.049 (2)	−0.009 (2)	0.002 (2)	−0.019 (2)
C6	0.054 (2)	0.062 (3)	0.063 (3)	−0.006 (2)	0.001 (2)	−0.025 (2)
C7	0.053 (2)	0.056 (2)	0.056 (3)	0.0005 (19)	−0.0031 (19)	−0.016 (2)
C7A	0.051 (2)	0.049 (2)	0.046 (2)	−0.0048 (18)	−0.0043 (17)	−0.0128 (18)
C8	0.063 (3)	0.058 (3)	0.058 (3)	0.003 (2)	−0.004 (2)	−0.024 (2)
C9	0.063 (3)	0.060 (3)	0.065 (3)	0.006 (2)	−0.008 (2)	−0.025 (2)
C10	0.084 (3)	0.058 (3)	0.078 (3)	0.011 (2)	−0.008 (3)	−0.028 (3)
C11	0.082 (4)	0.075 (4)	0.112 (5)	0.023 (3)	−0.021 (3)	−0.041 (3)
C12	0.057 (2)	0.045 (2)	0.045 (2)	−0.0020 (18)	−0.0097 (18)	−0.0132 (17)
C13	0.067 (3)	0.045 (2)	0.053 (2)	0.001 (2)	−0.015 (2)	−0.0169 (19)
C14	0.071 (3)	0.049 (2)	0.063 (3)	−0.011 (2)	−0.021 (2)	−0.011 (2)
C15	0.063 (3)	0.062 (3)	0.053 (2)	−0.008 (2)	−0.013 (2)	−0.006 (2)
C16	0.071 (3)	0.068 (3)	0.054 (3)	−0.013 (2)	0.002 (2)	−0.016 (2)
C17	0.076 (3)	0.057 (3)	0.051 (2)	−0.010 (2)	−0.004 (2)	−0.021 (2)
C18	0.085 (3)	0.065 (3)	0.065 (3)	−0.004 (2)	−0.009 (2)	−0.033 (2)
C19	0.076 (4)	0.096 (4)	0.086 (4)	−0.026 (3)	−0.013 (3)	−0.010 (3)
Cl1	0.0714 (11)	0.0803 (12)	0.0733 (11)	0.0080 (8)	0.0057 (8)	−0.0333 (9)
Cl1'	0.077 (4)	0.100 (4)	0.069 (3)	−0.018 (3)	0.010 (3)	−0.039 (3)

### Geometric parameters (Å, °)

**Table:**

S1—O2	1.419 (3)	C9—H9A	0.9700
S1—O1	1.427 (3)	C9—H9B	0.9700
S1—N1	1.679 (3)	C10—C11	1.517 (7)
S1—C12	1.739 (4)	C10—H10A	0.9700
N1—C2	1.401 (5)	C10—H10B	0.9700
N1—C7A	1.420 (5)	C11—H11A	0.9600
N3—C2	1.298 (6)	C11—H11B	0.9600
N3—C3A	1.387 (5)	C11—H11C	0.9600
C2—C8	1.488 (6)	C12—C17	1.394 (6)
C3A—C7A	1.389 (6)	C12—C13	1.410 (6)
C3A—C4	1.392 (6)	C13—C14	1.366 (6)
C4—C5	1.366 (7)	C13—C18	1.508 (6)
C4—H4A	0.9300	C14—C15	1.383 (7)
C5—C6	1.365 (7)	C14—H14A	0.9300
C5—Cl1'	1.750 (7)	C15—C16	1.382 (7)
C5—H5A	0.9300	C15—C19	1.493 (7)
C6—C7	1.390 (6)	C16—C17	1.362 (7)
C6—Cl1	1.747 (5)	C16—H16A	0.9300
C6—H6A	0.9300	C17—H17A	0.9300
C7—C7A	1.371 (6)	C18—H18A	0.9600
C7—H7A	0.9300	C18—H18B	0.9600
C8—C9	1.522 (6)	C18—H18C	0.9600
C8—H8A	0.9700	C19—H19A	0.9600
C8—H8B	0.9700	C19—H19B	0.9600
C9—C10	1.515 (6)	C19—H19C	0.9600
			
O2—S1—O1	119.5 (2)	C8—C9—H9B	109.4
O2—S1—N1	105.51 (18)	H9A—C9—H9B	108.0
O1—S1—N1	106.36 (18)	C9—C10—C11	112.0 (5)
O2—S1—C12	110.7 (2)	C9—C10—H10A	109.2
O1—S1—C12	108.22 (19)	C11—C10—H10A	109.2
N1—S1—C12	105.50 (18)	C9—C10—H10B	109.2
C2—N1—C7A	106.4 (3)	C11—C10—H10B	109.2
C2—N1—S1	126.9 (3)	H10A—C10—H10B	107.9
C7A—N1—S1	126.7 (3)	C10—C11—H11A	109.5
C2—N3—C3A	105.6 (3)	C10—C11—H11B	109.5
N3—C2—N1	112.4 (4)	H11A—C11—H11B	109.5
N3—C2—C8	124.4 (4)	C10—C11—H11C	109.5
N1—C2—C8	123.2 (4)	H11A—C11—H11C	109.5
N3—C3A—C7A	112.1 (4)	H11B—C11—H11C	109.5
N3—C3A—C4	128.0 (4)	C17—C12—C13	118.9 (4)
C7A—C3A—C4	119.9 (4)	C17—C12—S1	116.9 (3)
C5—C4—C3A	117.5 (4)	C13—C12—S1	124.2 (3)
C5—C4—H4A	121.3	C14—C13—C12	117.5 (4)
C3A—C4—H4A	121.3	C14—C13—C18	119.6 (4)
C6—C5—C4	121.5 (4)	C12—C13—C18	122.8 (4)
C6—C5—Cl1'	122.7 (4)	C13—C14—C15	124.2 (4)
C4—C5—Cl1'	114.0 (4)	C13—C14—H14A	117.9
C6—C5—H5A	119.2	C15—C14—H14A	117.9
C4—C5—H5A	119.2	C16—C15—C14	117.3 (4)
C5—C6—C7	122.8 (4)	C16—C15—C19	121.5 (5)
C5—C6—Cl1	119.8 (4)	C14—C15—C19	121.2 (5)
C7—C6—Cl1	117.4 (4)	C17—C16—C15	120.8 (4)
C5—C6—H6A	118.6	C17—C16—H16A	119.6
C7—C6—H6A	118.6	C15—C16—H16A	119.6
C7A—C7—C6	115.2 (4)	C16—C17—C12	121.4 (4)
C7A—C7—H7A	122.4	C16—C17—H17A	119.3
C6—C7—H7A	122.4	C12—C17—H17A	119.3
C7—C7A—C3A	123.0 (4)	C13—C18—H18A	109.5
C7—C7A—N1	133.5 (4)	C13—C18—H18B	109.5
C3A—C7A—N1	103.5 (3)	H18A—C18—H18B	109.5
C2—C8—C9	112.6 (4)	C13—C18—H18C	109.5
C2—C8—H8A	109.1	H18A—C18—H18C	109.5
C9—C8—H8A	109.1	H18B—C18—H18C	109.5
C2—C8—H8B	109.1	C15—C19—H19A	109.5
C9—C8—H8B	109.1	C15—C19—H19B	109.5
H8A—C8—H8B	107.8	H19A—C19—H19B	109.5
C10—C9—C8	111.0 (4)	C15—C19—H19C	109.5
C10—C9—H9A	109.4	H19A—C19—H19C	109.5
C8—C9—H9A	109.4	H19B—C19—H19C	109.5
C10—C9—H9B	109.4		
			
O2—S1—N1—C2	174.6 (3)	C4—C3A—C7A—N1	180.0 (4)
O1—S1—N1—C2	46.7 (4)	C2—N1—C7A—C7	−177.5 (5)
C12—S1—N1—C2	−68.1 (4)	S1—N1—C7A—C7	2.5 (7)
O2—S1—N1—C7A	−5.4 (4)	C2—N1—C7A—C3A	0.9 (4)
O1—S1—N1—C7A	−133.3 (3)	S1—N1—C7A—C3A	−179.1 (3)
C12—S1—N1—C7A	111.9 (4)	N3—C2—C8—C9	3.6 (7)
C3A—N3—C2—N1	1.0 (5)	N1—C2—C8—C9	−176.6 (4)
C3A—N3—C2—C8	−179.2 (4)	C2—C8—C9—C10	178.5 (4)
C7A—N1—C2—N3	−1.2 (5)	C8—C9—C10—C11	−179.4 (5)
S1—N1—C2—N3	178.8 (3)	O2—S1—C12—C17	−133.0 (3)
C7A—N1—C2—C8	179.0 (4)	O1—S1—C12—C17	−0.2 (4)
S1—N1—C2—C8	−1.0 (6)	N1—S1—C12—C17	113.3 (3)
C2—N3—C3A—C7A	−0.4 (5)	O2—S1—C12—C13	44.3 (4)
C2—N3—C3A—C4	179.3 (5)	O1—S1—C12—C13	177.1 (3)
N3—C3A—C4—C5	−179.1 (5)	N1—S1—C12—C13	−69.4 (4)
C7A—C3A—C4—C5	0.6 (7)	C17—C12—C13—C14	−1.5 (6)
C3A—C4—C5—C6	0.0 (8)	S1—C12—C13—C14	−178.7 (3)
C3A—C4—C5—Cl1'	−164.9 (4)	C17—C12—C13—C18	−177.9 (4)
C4—C5—C6—C7	0.1 (8)	S1—C12—C13—C18	4.9 (6)
Cl1'—C5—C6—C7	163.8 (4)	C12—C13—C14—C15	0.0 (7)
C4—C5—C6—Cl1	−179.9 (4)	C18—C13—C14—C15	176.5 (4)
Cl1'—C5—C6—Cl1	−16.3 (7)	C13—C14—C15—C16	1.0 (7)
C5—C6—C7—C7A	−0.9 (7)	C13—C14—C15—C19	−176.8 (4)
Cl1—C6—C7—C7A	179.2 (3)	C14—C15—C16—C17	−0.4 (7)
C6—C7—C7A—C3A	1.5 (6)	C19—C15—C16—C17	177.4 (5)
C6—C7—C7A—N1	179.6 (4)	C15—C16—C17—C12	−1.1 (7)
N3—C3A—C7A—C7	178.3 (4)	C13—C12—C17—C16	2.1 (7)
C4—C3A—C7A—C7	−1.4 (7)	S1—C12—C17—C16	179.5 (4)
N3—C3A—C7A—N1	−0.3 (5)		

### Hydrogen-bond geometry (Å, °)

**Table:**

*Cg*3 is the centroid of the C12–C17 ring.

**Table:**

*D*—H···*A*	*D*—H	H···*A*	*D*···*A*	*D*—H···*A*
C19—H19B···O2^i^	0.96	2.62	3.554 (7)	165
C4—H4A···Cg3^ii^	0.93	2.76	3.665 (8)	163

Symmetry codes: (i) *x*+1, *y*, *z*; (ii) −*x*+1, −*y*+1, −*z*+1.

## References

[bb1] Abdireimov, K. B., Mukhamedov, N. S., Aiymbetov, M. Zh. & Shakhidoyatov, Kh. M. (2010). *Chem. Heterocycl. Compd*, **46**, 941–946.

[bb3] Garuti, L., Roberti, M. & Cermelli, C. (1999). *Bioorg. Med. Chem. Lett.* **9**, 2525–2530. 10.1016/s0960-894x(99)00429-1 10498201

[bb4] Koči, J., Klimešová, V., Waisser, K., Kaustová, J., Dahse, H. M. & Möllmann, U. (2002). *Bioorg. Med. Chem. Lett.* **12**, 3275–3278. 10.1016/s0960-894x(02)00697-2 12392731

[bb5] Kubo, K., Inada, Y., Koharo, Y., Sugiura, Y., Ojima, M., Itoh, K., Furukawa, Y., Nashikawa, K. & Naka, T. (1993*a*). *J. Med. Chem.* **36**, 1772–1784. 10.1021/jm00064a011 8510105

[bb6] Kubo, K., Kohara, Y., Yoshimura, Y., Inada, Y., Shibouta, Y., Furukawa, Y., Kato, T., Nashikawa, K. & Naka, T. (1993*b*). *J. Med. Chem.* **36**, 2343–2349. 10.1021/jm00068a011 8360879

[bb7] Matsuno, T., Kato, M., Sasahara, H., Watanabe, T., Inaba, M., Takahashi, M., Yaguchi, S. I., Yoshioka, K., Sakato, M. & Kawashima, S. (2000). *Chem. Pharm. Bull.* **48**, 1778–1781. 10.1248/cpb.48.1778 11086914

[bb9] Sheldrick, G. M. (2008). *Acta Cryst.* A**64**, 112–122. 10.1107/S0108767307043930 18156677

[bb10] Stoe & Cie (1997). *STADI4* and *X-RED* Stoe & Cie, Darmstadt, Germany.

[bb11] Westrip, S. P. (2010). *J. Appl. Cryst.* **43**, 920–925.

